# Electrically Driven, Bioluminescent Compliant Devices for Soft Robotics

**DOI:** 10.1021/acsami.4c18209

**Published:** 2025-02-11

**Authors:** Kengo Kusama, Atsuro Oishi, Hitoshi Ueno, Akihide Yoshimi, Miki Nagase, Jun Shintake

**Affiliations:** † Department of Mechanical and Intelligent Systems Engineering, The University of Electro-Communications, 1-5-1 Chofugaoka, Tokyo 182-8585, Japan; ‡ Department of Anatomy, Kyorin University School of Medicine, 6-20-2 Shinkawa, Mitaka, Tokyo 181-0004, Japan; § Division of Cancer RNA Research, National Cancer Center Research Institute, 5-1-1 Tsukiji, Chuo, Tokyo 104-0045, Japan

**Keywords:** soft robotics, dielectric elastomers, bioluminescence, biohybrid, BRET

## Abstract

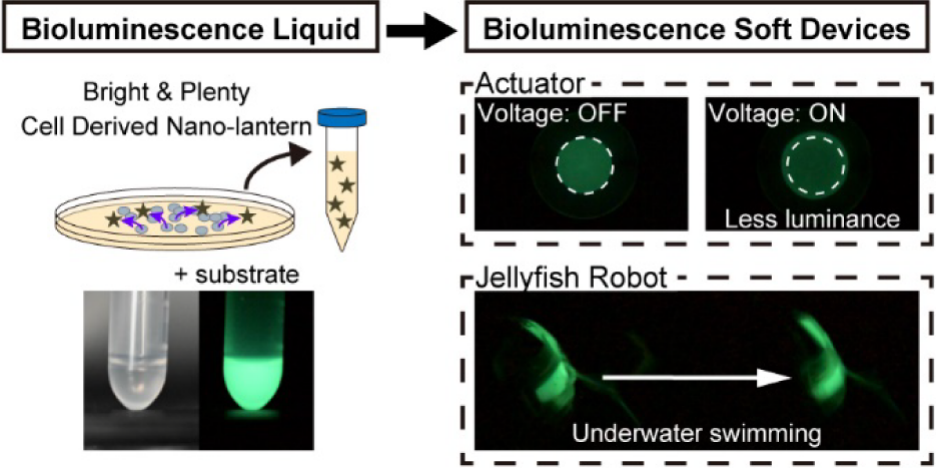

Soft robotics, a research field wherein robots are fabricated from compliant materials, has sparked widespread research interest because of its potential applications in a variety of scenarios. In soft robots, luminescence is an important functionality for communication and information transmission, and it is typically achieved through electroluminescence, which relies on synthetic substances activated by external electric sources, such as batteries. This paper focuses on the use of luciferase, a biologically derived luminescent enzyme, as a luminescent material. Bioluminescence, which is triggered by the luciferin–luciferase reaction, is highly energy-efficient, nontoxic, and eco-friendly. In this regard, a mammalian cell-derived secreted luciferase bioluminescent liquid was developed. This bioluminescent liquid is strongly bright, stable, freezable, and scalable for use as a soft robotic material. To investigate the applicability of this bioluminescent liquid to soft robotics, it was incorporated as an electrode in electrically driven soft actuators, sensors, and robots. Specifically, dielectric elastomer sensors (DESs) and dielectric elastomer actuators (DEAs) were fabricated and characterized using established fabrication processes. The resistivity of the bioluminescent liquid was found to be 448.1 Ω·cm. When the DES was subjected to uniaxial strain, it exhibited a linear response and large deformation of up to 200% strain, with a simultaneous luminance change of 27%. The DEA displayed an areal strain of 46.0% and a luminance change of 31% at an applied voltage of 3.4 kV. The waterproof bending DEA generated a tip angle of 21.8° at 10 kV and was applied to a jellyfish robot that could swim in water at a speed of 2.1 mm/s. The experimental results demonstrated the successful operation of these devices, validating the concept of energy-efficient, safe, and environmentally friendly bioluminescent soft robots.

## 1 Introduction

In recent years, there has been active research and development in the field of soft robotics, with a focus on the creation of robots from compliant materials.^1−5^ The inherent compliance enables soft robots to be adaptable, durable, and versatile in external environments while also making them safe for their surroundings. These features hold promise for diverse applications, including monitoring and rescue operations in natural environments and disaster zones,^6^ human–robot interactions,^7^ and human assistance.^8^ In soft robots, luminescence is an important functionality that enables communication between the robots and indicates their location and deformation status in their surrounding environments.^9−14^ Luminescence is also expected to enhance applications in biomedicine, such as the observation of biological tissues and biomonitoring, by leveraging soft robotics and advanced optical technologies.^15−17^ Most previous studies on soft robots have employed luminescence generated through electro-luminescence and chemical-luminescence,^9 −14^ which rely on synthetic substances and are often activated by external electric sources, such as batteries.

Herein, we focus on the use of luciferase, a biologically derived luminescent enzyme, as a luminescent material for soft robots. Bioluminescence, as well as fluorescence, is widely used in the field of life sciences and is induced by the luciferin–luciferase reaction, an energetically highly efficient chemical reaction.^18−22^ Since bioluminescence is derived from biological sources, it is nontoxic and environmentally friendly even when discarded. Furthermore, it does not require electricity, making it more energy-efficient, and unlike fluorescence, luminescence does not require light excitation.

Luminescent materials for soft robotics require two features: (i) sufficient brightness to be visible to the human eyes and (ii) productivity (quantity) and stability for use in soft robots. Obtaining visible light from luciferase has been challenging because of its low brightness; however, the brightness has been substantially increased to a level that is visible to the human eyes through the development of Nanolanterns by applying bioluminescence resonance energy transfer (BRET) technology, which utilizes bioluminescence emission energy to excite fluorescence protein.^23,24^ In the field of biology, some luciferases are used in secreted form for applications such as reporter gene assays,^25,26^ which could enable the mass production of bioluminescent liquids. In this study, we succeeded in producing a sufficient scale of visibly bright, mammalian cell-derived bioluminescent liquids by converting bright Nanolanterns into a secreted form, thereby enabling the creation of bioluminescent soft robots.

**Figure 1:**
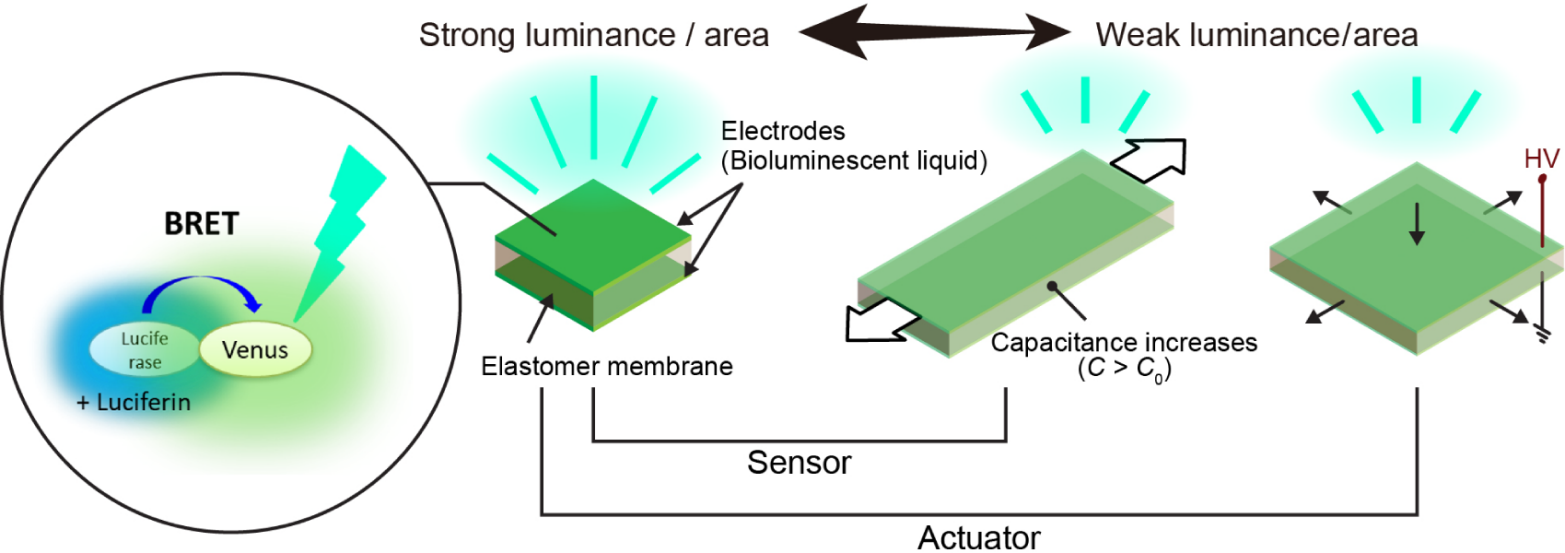
Principle of BRET in the Nanolantern and the structure and working principle of the DES and DEA.

Thereafter, we investigated the applicability of these bioluminescent liquids in soft robotics by incorporating them as electrodes in electrically driven soft robotic devices. We primarily focused on dielectric elastomer actuators (DEAs), which are a class of electroactive polymers.^27−35^ DEAs generally consist of a soft dielectric membrane sandwiched between compliant electrodes on both sides (Figure 1). When a voltage—typically in the kilovolt range—is applied, opposite electric charges accumulate on the electrodes, generating Maxwell stress (electrostatic force) between them. Maxwell stress (*p*) can be expressed as *p* = ε_0_ε*E*^2^, where ε_0_ is the vacuum dielectric constant, ε is the relative permittivity of the elastomer membrane, and *E* is the electric field generated between the electrodes. *E* can be expressed as *E* = *V*/*d*, where *V* is the applied voltage, and *d* is the thickness of the membrane. Maxwell stress triggers electrostatic actuation, causing the membrane to compress in thickness and expand in planar directions. DEAs exhibit large actuation strokes (exceeding 100% linear strain), fast response times (with a bandwidth of approximately 1 kHz), and high electrical and mechanical efficiency (theoretically around 90%).^33−35^ Additionally, the design of DEAs enables them to detect deformations by measuring changes in either the electrical resistance of the electrodes or the capacitance between them (Figure 1). DEAs utilized in sensing applications are known as dielectric elastomer sensors (DESs). Their combined actuation and sensing abilities make them highly promising candidates for bioluminescent soft robots.

To achieve the objective of this study, which is to investigate the applicability of bioluminescent liquids to DEAs and DESs, we first assessed the optical and electrical properties of the bioluminescent liquid. Next, we established fabrication processes to use the bioluminescent liquids as electrodes in DEAs and DESs, followed by characterized the resulting experimental samples. The results demonstrated that the bioluminescent DEAs and DESs operated successfully. As a proof of concept for soft robots, we developed a bioluminescent jellyfish robot and observed its underwater locomotion.

## 2 Results and Discussion

### 2.1 Characterization of the Nanolantern Based Secreted Bioluminescent Liquid

**Figure 2:**
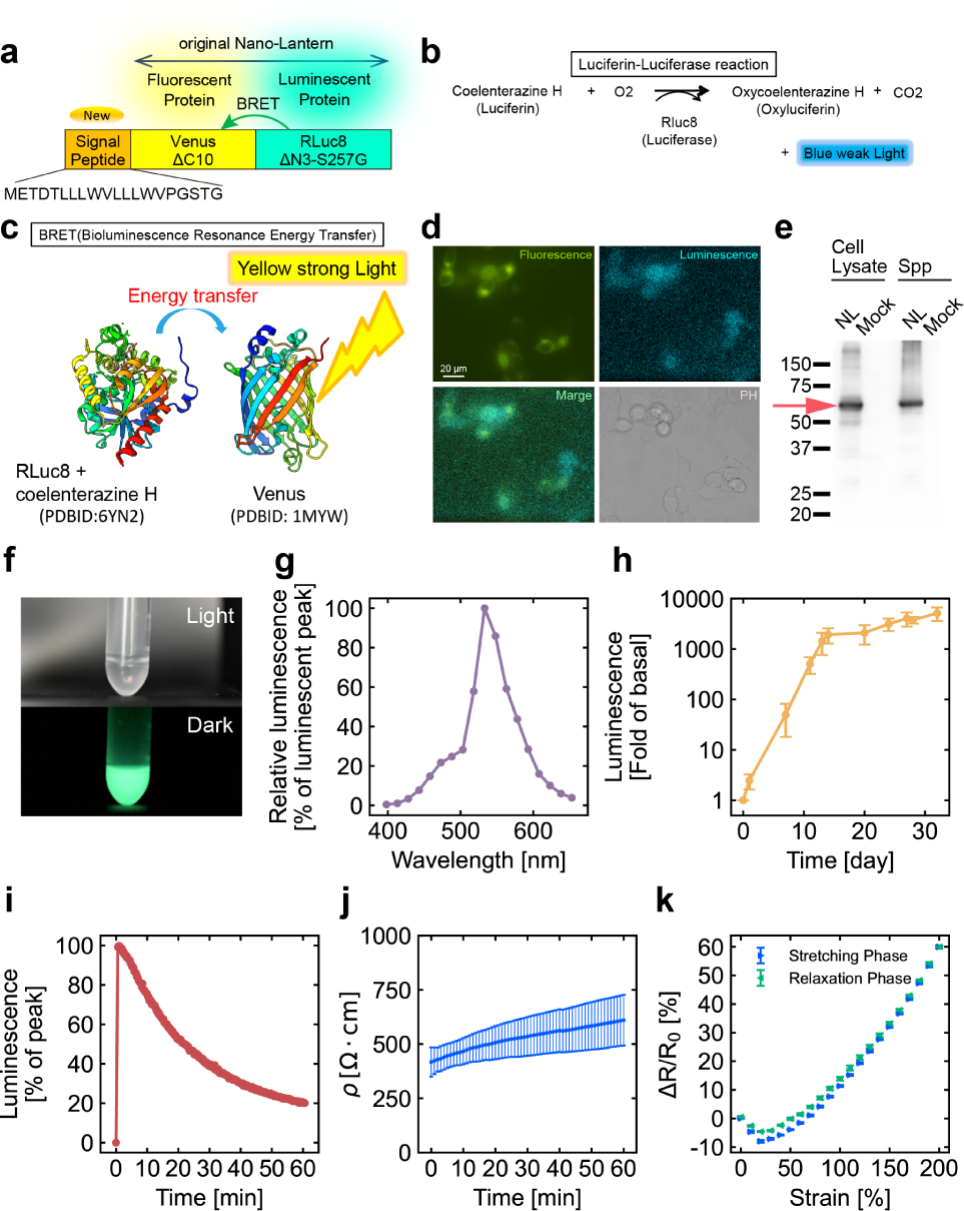
(a) Addition of a signal peptide for secretion to the Nanolantern. (b) Principle of luciferin–luciferase reaction in coelenterazine H + RLuc8. (c) Principle of BRET between RLuc8 and Venus. (d) Microscopic image of secreted Nanolantern plasmid-transfected cells. (e) Western blotting results of the Nanolantern (NL) in the transfected cells and supernatant (Spp). (f) Macroscopic image of the bioluminescent liquid under light and dark conditions. (g) Emission spectra of the secreted Nanolantern with coelenterazine H. (h) Kinetics of the accumulation of the secreted Nanolantern, as measured using the luminescent signal of the supernatant. (i) Kinetics of the luminescent signal of the secreted Nanolantern. (j) Resistivity of the luminescent liquid over time. (k) Resistance change of the luminescent liquid as a function of applied strain. The error bars in the data plots represent SD.

To develop a cell-derived brilliant luminescent liquid, we selected Nanolantern, which is a highly bright fusion protein of the fluorescent Venus and luminescent RLuc8 variants, using BRET technology as an essential luminescent protein and modified it for secretion outside cells (Figure 2a).^23^ The luminescence mechanism operates as follows: The luminescent substrate luciferin, specifically coelenterazine H, undergoes an oxidation reaction catalyzed by the luciferase RLuc8. During this process, carbon dioxide is released, and the oxidized luciferin transitions to an excited state, emitting blue light as it returns to the ground state (Figure 2b). However, oxidized luciferin exhibits low luminescence efficiency, which results in weak blue-light emission.^36,37^ In the Nanolantern, the energy of the luminescent substrate oxidized by modified RLuc8 is transferred to the adjacent fluorescent protein Venus, which has high luminescence efficiency and emits strong yellow light, a phenomenon called BRET (Figure 2c).^23^ Furthermore, by adding a signal peptide of a secretory protein to the N-terminus of the Nanolantern, we modified it to be synthesized intracellularly and then secreted into the cell supernatant (Figure 2a). This construct was transfected into HEK293T cells, and the intracellular expression of the Nanolantern was confirmed through both fluorescence and luminescence microscopy analyses (Figure 2d). The intracellular expression of the Nanolantern and its secretion into the cell supernatant were confirmed by Western blotting at the appropriate molecular weight (Figure 2e). The results revealed that the Nanolantern was produced in the cells and successfully secreted to the supernatant. Next, the electrical characteristics of the bioluminescent liquid were evaluated. Initially, the resistivity of the base liquid medium was measured to be ∼200 Ω·cm (Figure S1). Since the medium had high conductivity and could allow cells to be cultured for several weeks while continuously secreting, it was used to create a bioluminescent liquid. The secreted luminescent liquid was weakly but obviously visible to the naked eye in complete darkness, and 2.5 s exposure allowed capturing very bright emission light by commercially available digital camera (Figure 2f). The luminescence spectrum of the Nanolantern-containing supernatant displayed a peak around 530 nm, a feature of the original Nanolantern (Figure 2g).^23^

The luminescence intensity of the Nanolantern that was secreted into the cell supernatant over time increased logarithmically for up to 2 weeks and then continued to increase gradually without decreasing (Figure 2h). Therefore, the bioluminescent liquid was collected for use after about 4 weeks. After the addition of a substrate, coelenterazine H, the luminescence intensity of the obtained bioluminescent liquid peaked within 1 min and then gradually declined (Figure 2i). The observed decay over time was primarily attributed to the consumption of luciferin, which is significantly influenced by the concentration of luciferase.^24,38,39^ To examine the relationship between luminescence intensity and luminescence decay, various concentrations of bioluminescent liquid containing Nanolantern and coelenterazine H were analyzed. A 25 μM coelenterazine H with 100% bioluminescent liquid was identified as providing sufficient substrate (Figure S2a) to achieve maximal overall brightness, albeit with a slightly faster decay than 10% bioluminescent liquid (Figure S2b). Consequently, subsequent experiments were conducted using 25 μM coelenterazine H with 100% bioluminescent liquid, using appropriate controls to account for this time-dependent decay.

Figure 2j shows the time-dependent variation in the resistance of the bioluminescent liquid. The change in the resistance of the bioluminescent liquid was negligible for at least 0–60 min, confirming the applicability of this liquid to the subsequent study. Figure 2k shows the resistivity change as a function of uniaxial strain. The resistivity of the bioluminescent liquid was initially 448.1 ± 135.0 Ω·cm. The resistance tended to increase nonlinearly with strain. However, the resistance decreased at 0%–50% strain owing to the plastic deformation of the experimental samples. The results of the analysis of the electrical characteristics indicate that the bioluminescent liquid is highly conductive.

**Figure 3:**
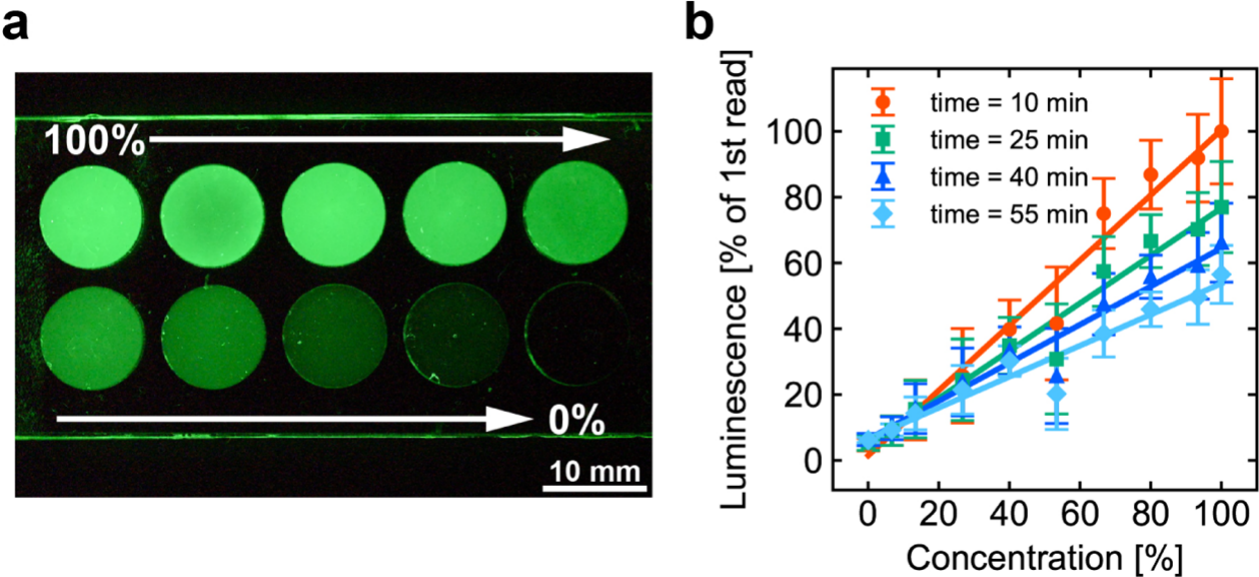
Calibration experiments. (a) Luminescence at varying concentrations. (b) Calibration curve of concentration versus luminescence intensity over time. The error bars represent SD.

In the subsequent experiments, the image of the samples was converted to a grayscale, and the grayscale values were used to quantify the luminance. To ensure the quantitative accuracy of this method, a calibration curve was generated from the relationship between bioluminescent liquid concentration and luminance. Ten different concentrations of the bioluminescent liquid (Table S1) were applied to a grooved plate, as shown in Figure 3a, and the luminance was recorded. Figure 3b shows the concentration–luminance relationship at the start of the experiment (10 min after the addition of coelenterazine H) and 15, 30, and 45 min later, along with an approximate straight line, or a calibration curve, obtained using the least-squares method. The coefficients of determination for the approximate lines were all greater than 0.94, indicating a strong linear relationship between the bioluminescent liquid concentration and luminance and confirming the quantitative validity of the luminance measurement method.

### 2.2 Bioluminescent Soft Robotic Devices

We established a fabrication process for the bioluminescent DES and DEA and the waterproof bending DEA (see Section 4 for more details). The bending DEA was used for the jellyfish robot.

**Figure 4:**
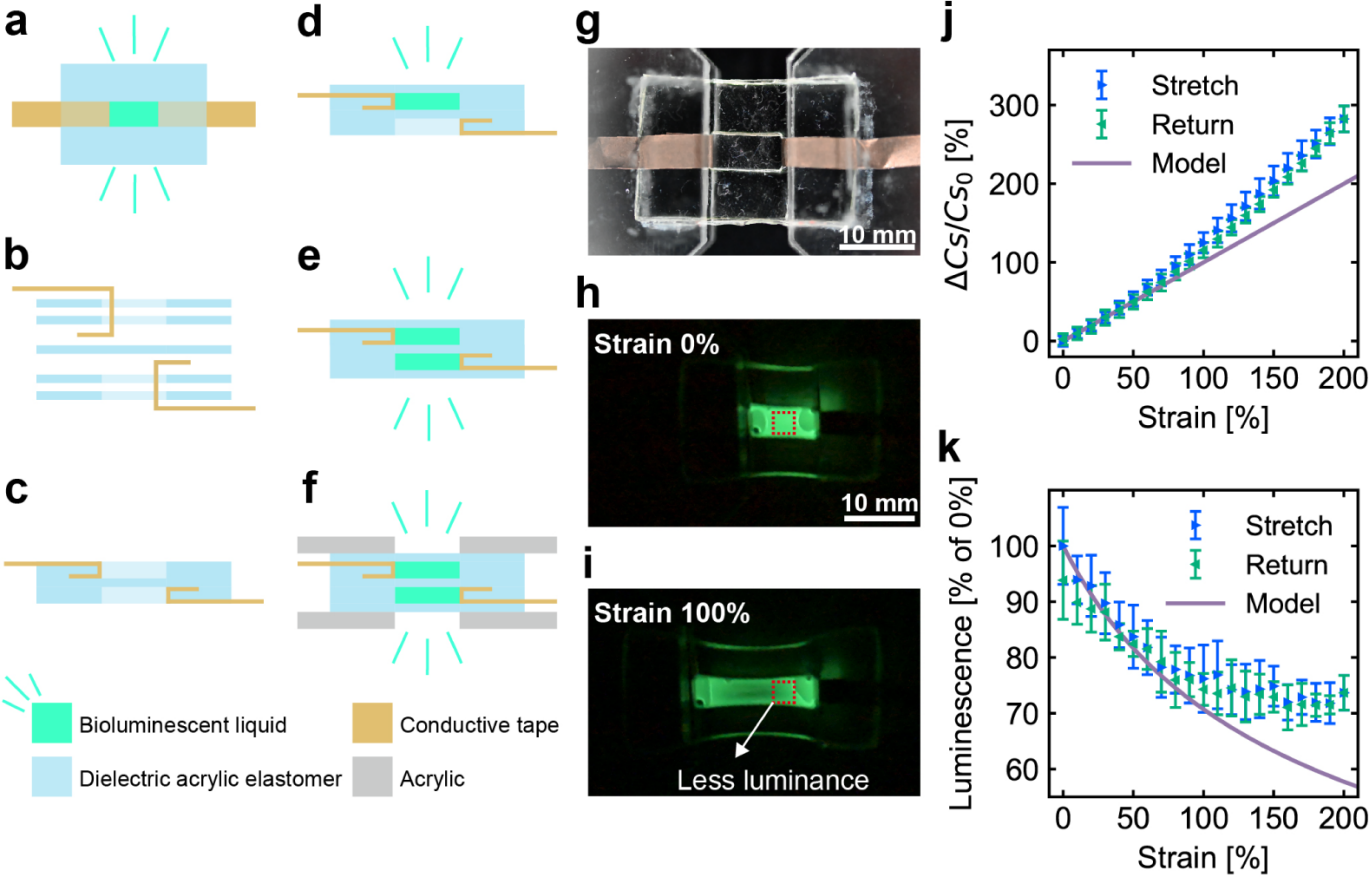
(a) Top view and (b–f) fabrication process of the DES. (b) Positioning and (c) lamination of the dielectric acrylic elastomer and conductive tape. (d) Injection and sealing of the bioluminescent liquid. (e) Injection of the bioluminescent liquid into the opposite side and sealing it. (f) Insertion of the DES into an acrylic holder. (g) DES under room light conditions. (h) Reference and (i) stretched DES states under dark conditions. (j) Capacitance and (k) luminance changes of the DES as functions of the applied strain. The error bars in the data plots represent SD.

We first characterized the DES. The DES (Figure 4) was made by laminating dielectric acrylic elastomers to create two bioluminescent liquid-filled layers (Figure 4a–f). The capacitance of the fabricated DES (Figure 4g) at zero applied strain was 448.1 ± 135.0 pF. However, the capacitance changed when the DES was subjected to uniaxial strain (Figure 4h,i), as shown in Figure 4j. The capacitance increased linearly with strain, reaching ∼280% change at 200% strain. The gauge factor was 1.25 at 100% strain and 1.41 at 200% strain. Furthermore, the DES was found to change its luminance in response to deformation. The relationship between strain and luminance is depicted in Figure 4k. The luminance tended to decrease nonlinearly as the strain increased. The DES comprises an elastomer that can be considered incompressible materials that maintain a constant volume despite deformation. Consequently, when a uniaxial strain is applied to the DES, compression occurs in other directions. This compression-induced reduction in the thickness of the DES leads to a corresponding decrease in luminance. The luminance change decreased by −24% at 100% strain and by −26% at 200% strain. The gauge factor was −0.24 at 100% strain and −0.13 at 200%. Changes in capacitance and luminance were generally in agreement with the model (see Supporting Information for more details). The results demonstrate that the DES was able to function as a sensor for detecting strain based on both capacitance and luminance. This indicates that the bioluminescent liquid is effective as an electrode material for capacitive strain sensors.

**Figure 5:**
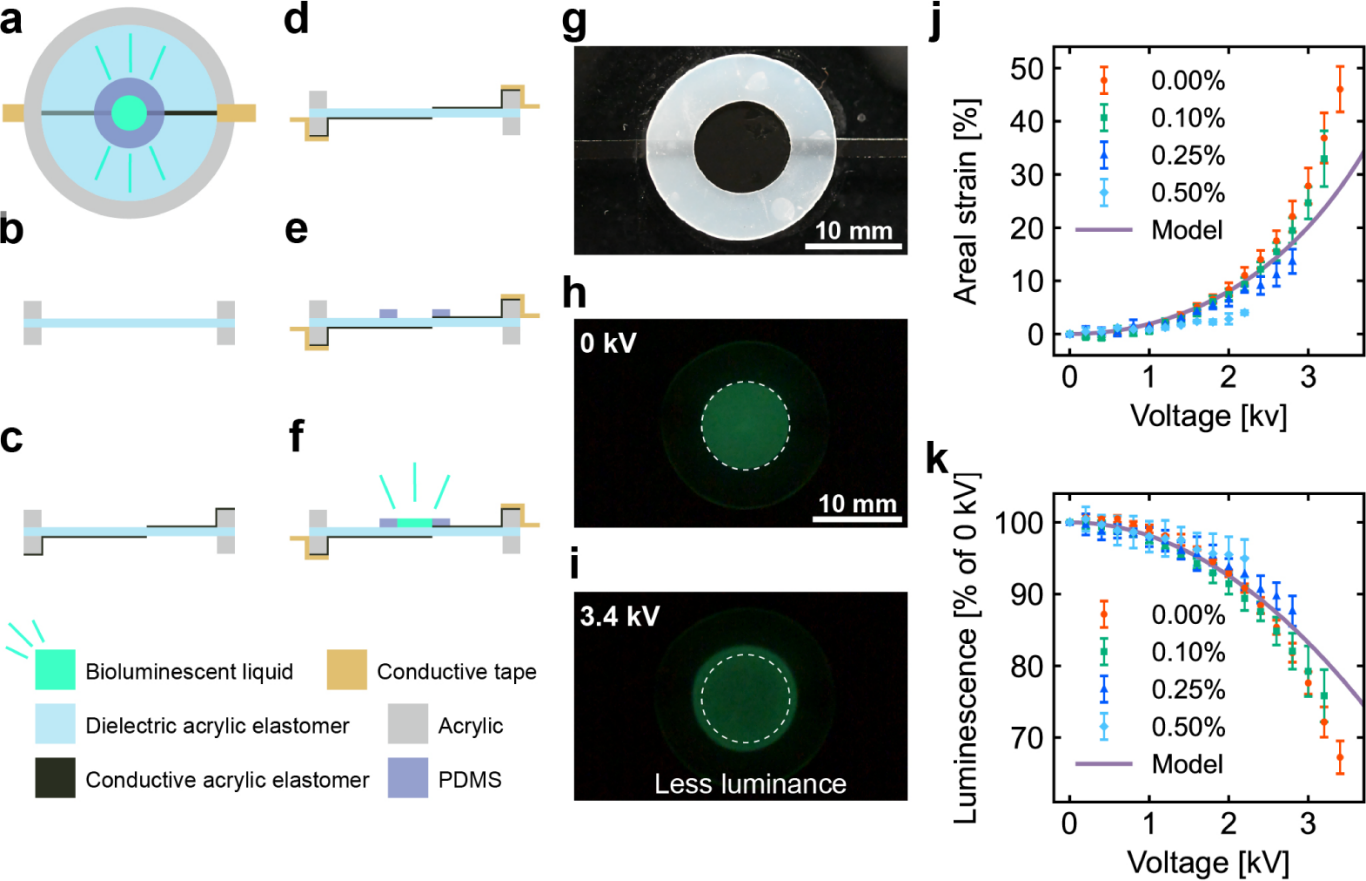
(a) Top view and (b–f) fabrication process of the DEA. (b) Pretensioning of the circular dielectric acrylic elastomer to 250% and holding in an acrylic frame. (c) Attachment of the conductive acrylic elastomer. (d) Peeling off the protective film and attaching the conductive tape. (e) Attachment of a ring-shaped silicone sheet. (f) Pouring of the bioluminescent liquid. (g) DEA under room light conditions. (h) Reference and (i) activated states of the DEA under dark conditions. (j) Areal strain and (k) luminance change of the DEA as functions of the applied voltage. The error bars in the data plots represent SD.

We subsequently assessed the actuation characteristics of the DEA. The DEA (Figure 5) was fabricated by attaching a ring-shaped silicone mold onto a prestrained dielectric acrylic elastomer and then pouring the bioluminescent liquid into the mold to form an electrode (anode) (Figure 5a–f). The cathode was made of a conductive acrylic elastomer. An agarose gel was added to the bioluminescence liquid for shape stabilization. Gel concentrations of 0%, 0.10%, 0.25%, and 0.50% were formulated. Applying a high voltage to the fabricated DEA (Figure 5g) increased the areal strain of the electrodes and decreased the luminance, as depicted in Figure 5h,i. The areal strain and luminance change as functions of the applied voltage are shown in Figure 5j,k, respectively. The DEA uses an elastomer as the dielectric membrane. As previously mentioned, this type of material maintains a constant volume even when deformed. The elastomer is positioned between a bioluminescent liquid electrode and a conductive acrylic elastomer electrode. When a voltage difference is applied across the electrodes, deformation occurs in both the planar and thickness directions. Consequently, the thickness of the bioluminescent liquid electrodes also changes while their volume remains constant. Given that the luminance of the electrodes is proportional to their thickness, planar deformation reduces luminance. The DEA with an electrode containing a 0% gel concentration exhibited a maximum areal strain of approximately 46.0% and a maximum luminance change of ∼31% at an applied voltage of 3.4 kV. The areal strain increased in proportion to the square of the applied voltage because the Maxwell stress is proportional to the square of the voltage. The change in luminance diminished proportionally to the square of the applied voltage. This is due to the decrease in electrode thickness caused by the increase in areal strain; since the thickness is inversely proportional to areal strain, luminance is expected to decrease quadratically. The changes in areal strain and luminance were consistent with the model (see Supporting Information for details), but the measured values deviated more significantly from the model as the voltage approached its maximum. This discrepancy may occur because the model assumes constant film thickness, whereas in reality, a thinner film leads to a stronger electric field. We also observed an electrical breakdown of the DEA membrane at voltages above 3.4 kV for all gel concentrations. This resulted from the high electric field intensity caused by the prestrain on the dielectric elastomer and the thinning of the dielectric elastomer due to actuation. Figure 5j shows that the areal strain of the electrodes increased as the gel concentration decreased. This is because high gel concentrations increase the rigidity of the electrodes, which inhibits deformation. Nevertheless, this result demonstrates that our bioluminescent liquid can provide electrostatic actuation of DEAs by serving as their electrodes even in the agarose gel.

**Figure 6:**
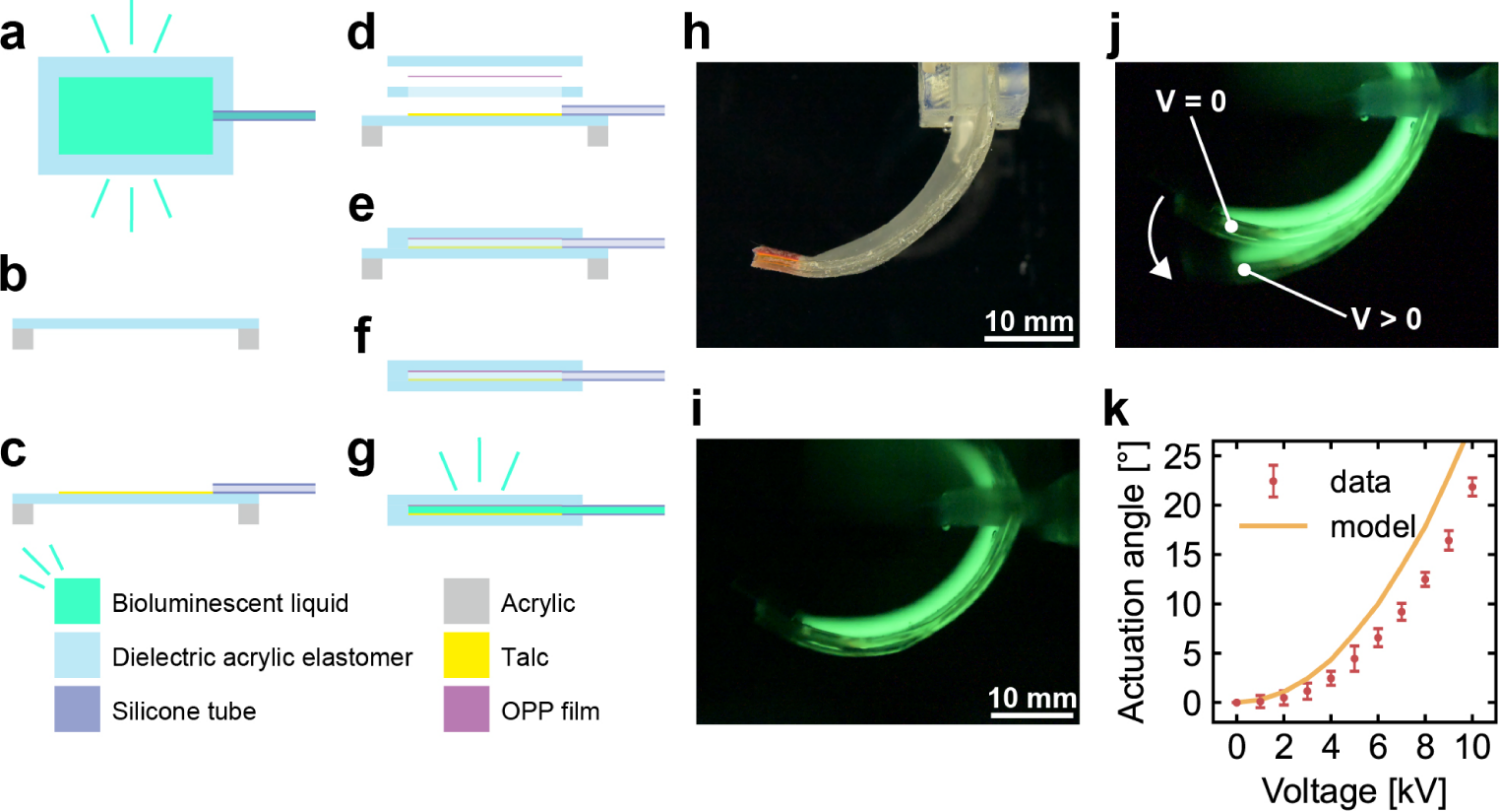
(a) Top view and (b–g) fabrication process of the waterproof bending DEA. (b) Pretensioning of the dielectric acrylic elastomer. (c) Application of talc and placement of the silicone tubing. (d) Placement of the dielectric acrylic elastomer and OPP film to overlap the talc-applied surface and (e) their lamination. (f) Removal of excess material and (g) injection of the bioluminescent liquid. (h) Waterproof bending DEA in room light (underwater). (i) Reference and (j) activated states of the bending DEA under dark conditions (underwater). (k) Tip angle of the waterproof bending DEA as a function of the applied voltage. The error bars in the data plots represent SD.

The waterproof bending DEA (Figure 6) was made by laminating a dielectric acrylic elastomer, a silicone tube, and a flexible, nonstretchable oriented polypropylene (OPP) film and then injecting the bioluminescent liquid into the resulting layer (Figure 6a–g). Talc powder was used at the injection site to minimize the adhesive strength of the dielectric acrylic elastomer. The waterproof bending DEA utilizes the bioluminescent liquid as the anode and the surrounding environmental water as the cathode. Figure 6h displays the fabricated waterproof bending DEA. This actuator is designed to exhibit actuation strokes underwater to generate thrust forces in swimming robots. Figure 6i illustrates how the bending DEA emits light in the dark, while Figure 6j illustrates its operation. The image of the actuator was captured with a 10 s exposure, wherein the DEA was captured with the voltage turned off for 5 s and then with the voltage turned on for 5 s. Given the minor actuated strain of the DEA in this bending configuration, the change in brightness per unit area is minimal and visually imperceptible (Figure 6j). However, a key advantage of this luminescent bending DEA is its ability to clearly exhibit deformation states from an external perspective in dark environments. This feature is well-suited for visualizing out-of-plane motions and tracking robot movements using the same actuator.

The tip angle displacement of the actuator increased as the applied voltage increased, reaching 21.8° at 10 kV (Figure 6k). The actuation angle was in good agreement with the model (see Supporting Information for details). This result implies that the bioluminescent liquid can be used both in the air and in aquatic environments.

### 2.3 Jellyfish Robot

**Figure 7:**
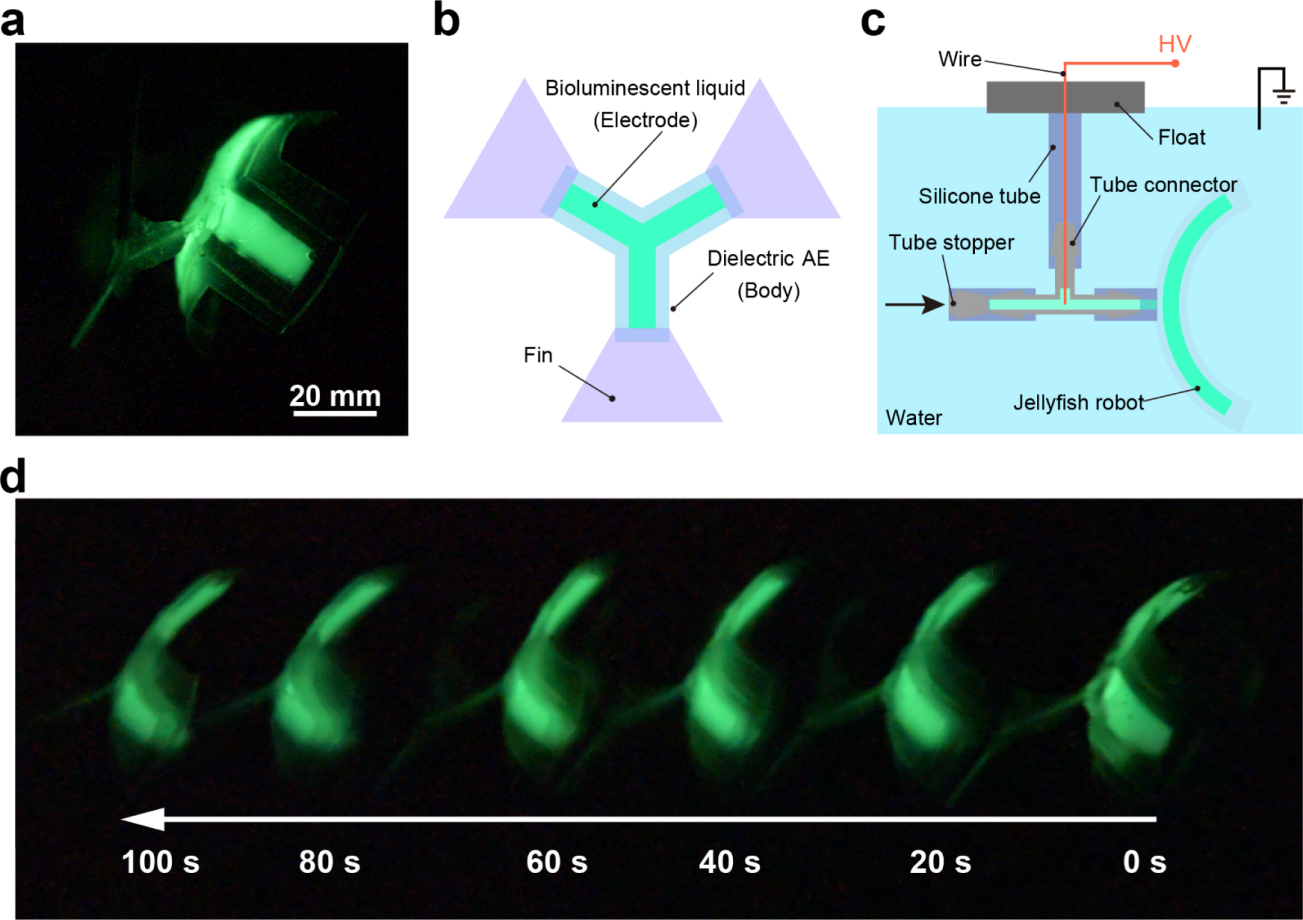
(a) Appearance of the jellyfish robot. (b) Top view of the robot. (c) Schematic of the experimental setup. (d) Observed swimming motion of the robot.

To investigate the potential application of soft robots based on the bioluminescent liquid, we developed a jellyfish robot that utilizes the waterproof bending DEA (Figure 7a, see Section 4 for more details). Figure 7b depicts the top view of the robot. As shown in Figure 7c, the robot was connected to a float using a silicone tube and connectors and placed in a water tank filled with tap water. It was electrically connected to a voltage source at the end of the float through an enamel wire that passed through the tube and the bioluminescent liquid. The tube connectors extended in three directions. The bioluminescent liquid was injected from arrow direction (neither the jellyfish robot side nor the float side) and was sealed from the outside with a tube stopper. The jellyfish robot was powered by a 10 kV, 0.5 Hz square wave and swam at a speed of about 2.1 mm/s (Video S1). As shown in Figure 7d, the jellyfish robot emitting the light clearly indicates its position in the dark during the swimming, which demonstrated that our bioluminescent liquid provided the luminous function to the soft robotics.

## 3 Conclusion

In this study, we succeeded in producing strongly bright, stable, freezable, and scalable bioluminescent liquid. Then we characterized its features and demonstrated the applicability of a bioluminescent liquid to soft robotics by employing it as a luminescent electrode to develop electrically driven soft sensors, actuators, and robots. The experimental results revealed that these luminescent devices operated successfully, thereby proving the broad applicability of bioluminescence to the engineering field and validating the concept of energy-efficient, nontoxic, and environmentally friendly bioluminescent soft robots.

Future concerns will involve improving the luminescence procedure. In this study, a bioluminescent liquid was prepared in a luminescent state by mixing a Nanolantern and coelenterazine H during the device fabrication stage. The intensity of the bioluminescent liquid decays over time; therefore, fabrication and experiments must be completed quickly. Moreover, the storage stability of the device is poor, but this issue can be solved by further optimizing the device geometry and controlling the progression of the luminescence reaction, as well as the optimization of bioluminescent liquid. The luminescence intensity can be improved by designing structures wherein the Nanolantern and coelenterazine H are mixed through the actuation of the device or by applying strain, thereby inducing luminescence. Furthermore, the luminescence can be enhanced by utilizing a flow path that allows the bioluminescent liquid inside the element to be replaced. Additionally, the reaction rate of bioluminescence can be controlled by adjusting environmental factors such as solution concentration, temperature, enzyme quantity, and oxygen and nitrogen levels. These reaction rate control methods allow for the activation of luminescence at any time. Another concern is improving the electrical properties of the bioluminescent liquid, particularly its conductivity. However, since the resistance of the bioluminescent liquid is determined by the culture medium, it can be adjusted by changing the electrical properties of the culture medium.

## 4 Experimental Section

### 4.1 cDNA and Material

The cDNA-encoding full-length Nanolantern was modified by performing polymerase chain reaction (PCR) on Nanolantern(cAMP-1.6)/pcDNA3, which was a gift from Takeharu Nagai (Addgene plasmid #53594; http://n2t.net/addgene:53594;RRID:Addgene_53594). The cDNA-encoding secreted Nanolantern was created by integrating a signal peptide (METDTLLLWVLLLWVPGSTG) for secretion^40^ into the N-terminal regions of the Nanolantern via a standard PCR, as previously described.^41^ Coelenterazine H was purchased from Wako (Japan). The HEK293T cells were gifted by Dr. Ralf Jockers.^42^

### 4.2 Cell Culture and Transfection

Cell culture and transient transfection were performed as previously described.^43^ Briefly, the HEK293T cells were grown in complete DMEM (Wako 043-30085) supplemented with 10% (v/v) fetal bovine serum (FBS) and maintained at 37 °C (95% O_2_, 5% CO_2_). The transient transfection of the cells was performed with a PEI MAX (PSI 27465-1) reagent according to the supplier’s instructions.

### 4.3 Preparation and Specification of the Bioluminescent Liquid

First, 10 μg of plasmids containing the secreted Nanolantern was transfected into 80% confluent HEK293T cells in a 10 mL dish (approximately 7 × 10^6^ cells). Next, 24 h after transfection, the medium was completely aspirated and substituted with 10 mL of phenol red-free DMEM (Wako 040–30095) without FBS. The medium was maintained for 4 weeks without substitution, and the accumulated secreted Nanolantern-containing medium was collected after centrifugation (400 *g* × 5 min) to spin down the floating or dead cells. The collected secreted Nanolantern-containing medium was frozen at −20 °C until use. To prepare the actuator and conduct the experiments, the frozen bioluminescent liquid was quickly thawed in the hand, and then coelenterazine H was added at a final concentration of 25 μM. The macroscopic image of the bioluminescent liquid under light and dark conditions (Figure 2c) was captured for 2.5 ms exposure by a digital camera (Nikon, Z5).

### 4.4 Characterization of the Bioluminescent Liquid

Western blotting was performed as previously described.^44^ Briefly, denatured proteins in Laemmli sample buffer were resolved in 10% SDS-PAGE gels, transferred to PVDF membranes, and immunoblotted with antibodies against a primary antibody, anti-GFP antibody (1:20000 dilution, Proteintech; 66002-1-Ig). Immunoreactivity was revealed using Goat Anti-Rabbit IgG (1:10000 dilution, Jackson ImmunoResearch; 111-035-144) with Clarity Western ECL substrates (BIO-RAD; 170-5061) and then visualized using an ImageQuant LAS 4000 imaging system (GE Healthcare, USA).

The live cell image of the secreted Nanolantern-transfected cells was captured using the following procedures. HEK293T cells that transiently expressed the secreted Nanolantern were seeded onto a sterile poly-l-lysine-coated (Wako, 339-30753) cover glass (Matsunami, 83-0217) with a silicone chamber (ibidi, ib81201) 1 day after transfection. The next day, the medium was substituted into phenol red-free DMEM (Wako, 040-30095) for 2 h, and then the cells were examined under fluorescent conditions using a U-YFP mirror unit (Olympus). After adding 5 μM (final concentration) coelenterazine H, the luminescence images were acquired using an objective lens UPLXAPO100XO (Olympus) with an inverted microscope IX83 (Olympus, Japan) and an electron-multiplying charge-coupled device camera ImagEM X2 (Hamamatsu Photonics) for a 5 min exposure.

The secretion kinetics of the luminescent liquid was measured as follows. Briefly, 80% confluent HEK293T cells seeded in 24-well plates were transiently transfected with 500 ng of the secreted Nanolantern or mock plasmids (pcDNA3.1+) in triplicate. Twenty-4 h after transfection, the medium was substituted with 500 μL of phenol red-free DMEM (Wako, 040–30095) and maintained at 37 °C in a 5% CO_2_ incubator. The 5 μL medium was collected at designated time points and freezed until measurement. The emission spectrum and luminescent signal of the secreted Nanolantern (Figure 2e,f) were acquired using TECAN SPARK10 M (Tecan Group, Ltd., Switzerland).

For the measurement of decaying luminescent signal of bioluminescent liquid (Figure 2g), the luminance of the bioluminescent liquid (around 4 weeks secretion) after the addition of 25 μM coelenterazine H (final concentration) was monitored with OD3 filter by TECAN SPARK10 M (Tecan Group, Ltd., Switzerland).

A dielectric acrylic elastomer (3M, VHB 4905) was cut into two 30 mm × 20 mm pieces and three 30 mm × 20 mm pieces with a 10 mm × 5 mm hole using a laser cutting machine (Trotec, Speedy 300). These pieces were then sequentially stacked to create a space of 10 mm × 5 mm × 1.5 mm. The bioluminescent liquid was sealed in this space and electrically connected to an LCR meter (GW Instek, LCR-6002) with conductive tape (Teraoka Corporation, double-sided conductive copper foil tape) to measure its change in resistance over time. The change in the resistance of the bioluminescent liquid under strain was measured by fixing a sample of the same shape on a motorized stage (Zaber, X-LRT1000DL) using an acrylic fixture and applying tension. The tensile speed was set to 10 mm/s.

### 4.5 Acquisition of the Calibration Curve

The bioluminescent liquid was diluted with water to create 10 concentrations of the liquid (Table S1). The prepared bioluminescent liquid was distributed on a plate with 10 grooves made by a 3D printer (Formlabs, Form3) and placed in an environment shielded by a darkroom (Azwan, table-top darkroom BBX-01X) and a blackout curtain (ETSUMI, blackout curtain for darkrooms). The luminescence was measured using a camera (Nikon, Z5, lens: AF-S Micro NIKKOR 60 mm f/2.8G ED). The camera settings were an exposure time of 2.5 s, an F value of 3.2, and an ISO sensitivity of 25600. Photographs were captured at 0, 5, 10, 15, 20, 25, 30, and 45 min from the start of the experiment, resulting in a total of 8 time points. This measurement was repeated three times, and the average was recorded.

### 4.6 Fabrication and Characterization of the DES

The DES was fabricated by laminating seven layers of dielectric acrylic elastomers (3M, VHB 4905) cut into 30 mm × 20 mm shapes using the laser cutting machine (GW Instek, LCR-6002). Layers 2, 3, 5, and 6 each had a 10 mm × 5 mm hole. First, layers 2, 3, 4, 5, and 6 were laminated to form two 10 mm × 5 mm × 1 mm spaces. During this step, conductive copper foil tape (Teraoka Seisakusho, double-sided) was attached to establish an electrical connection between the space and the outside. This space was filled with the bioluminescent liquid and then sealed by attaching layers 1 and 7, creating an electrode. Finally, the DES was sandwiched between acrylic holders.

The DES was fixed to a motorized stage (Zaber, X-LRT1000DL) using an acrylic holder, and the uniaxial strain was applied to measure changes in capacitance using the LCR meter (GW Instek, LCR-6002). The motorized stage speed was set at 1 mm/s. Changes in luminance were captured using a camera (Nikon, Z5, lens: AF-S Micro NIKKOR 60 mm f/2.8G ED). The camera was set to an exposure time of 2.5 s, f/3.0, and an ISO sensitivity of 25600 to achieve long-exposure noise reduction and high-sensitivity noise reduction. Experiments were conducted in a dark room shielded by a blackout curtain. The luminance calculation method is explained in Supporting Information. During this experiment, three samples were measured, and the average was recorded.

### 4.7 Fabrication and Characterization of the DEA

A dielectric acrylic elastomer (3M, VHB 4905) was cut into circular shapes using the laser cutting machine (Trotec, Speedy 300), prestretched by 250% on a stretcher, and held between a circular acrylic frame (inner diameter: 45 mm; outer diameter: 55 mm). A conductive acrylic elastomer (Adhesives Research, ARcare 90366) was laser-cut into a composite of a 10 mm diameter circle and a 25 mm × 2 mm rectangle and attached to one side of the dielectric acrylic elastomer layer. A second 25 mm × 2 mm rectangular conductive acrylic elastomer was attached to the opposite side. After removing the protective film from the conductive acrylic elastomer, conductive foil tape (Teraoka Seisakusho, conductive aluminum foil adhesive tape) was attached to establish an electrical connection. Next, two silicone elastomers (Smooth-On, Ecoflex 00-10 and Ecoflex 00-30 elastomers) were combined in a 1:1 weight ratio and stirred with a stirrer (THINKY, ARE-310) at 2000 rpm for 10 min. The mixture was blade-cast with a film applicator (TQC Sheen B.V., AB4220) and a universal film applicator (Zehntner, ZUA 2000) to produce silicone sheets with a thickness of 1 mm. The blade height was 1.5 mm, and the silicone was cured at 80 °C for 20 min. The silicone sheet was laser-cut into a circular shape with an inner diameter of 10 mm and an outer diameter of 20 mm and attached to the dielectric acrylic elastomer layer. Finally, the bioluminescent liquid was injected into the circular silicone sheet.

The luminescent agarose gel was made by combining Agarose S (Nippon Gene 318-01195) with the bioluminescent liquid to stabilize its shape. The gel, which contained 1% W/V agarose/distilled water, was melted by heating it in a block bath (Major Science, Mini-Block Bath MD-MINI) at 95 °C, and then four concentrations of the gel (0%, 0.10%, 0.25%, and 0.50%) were mixed with the bioluminescent liquid. To prevent the bioluminescent liquid from doming, 0.1% surfactant (Dow, Triton X-100) was introduced into each luminescent electrode.

For characterization, the DEA was operated with a DC voltage input from a DC–DC converter (XP-Power, CB101) controlled by a function generator (Matsusada Precision, eK-FGJ) and a stabilized power supply (KIKUSUI, PMX32–2QU). Changes in the surface distortion and luminance of the bioluminescent electrode were measured using a camera (Nikon, Z5, lens: AF-S Micro NIKKOR 60 mm f/2.8G ED) under an applied voltage. The camera was set to an exposure time of 2.5 s, f/3.2, and an ISO sensitivity of 25600 to achieve long-exposure noise reduction and high-sensitivity noise reduction. Experiments were conducted in a darkroom (As One, BBX-01X table-top darkroom) shielded by a blackout curtain (ETSUMI, blackout curtain for darkrooms). The method for calculating the luminance is provided in Supporting Information. During this experiment, three samples were measured, and the average was reported.

### 4.8 Fabrication and Characterization of the Waterproof Bending DEA

A dielectric acrylic elastomer (3M, VHB 4905) was laser-cut into three different shapes: 60 mm × 25 mm, 40 mm × 20 mm, and 40 mm × 20 mm with a 30 mm × 10 mm hole. First, the 60 mm × 25 mm dielectric acrylic elastomer was pretensioned by 110% in the uniaxial direction using a stretcher. Thereafter, talc was applied to the 30 mm × 10 mm area, and a silicone tube (MonotaRO, MGJG-1 × 2) for liquid injection was placed. The two 40 mm × 20 mm dielectric acrylic elastomers were laminated, and an OPP film (TOYOBO, PYLEN) with the same shape was attached inside the 30 mm × 10 mm hole. This film restricts actuation on one side of the device and results in bending actuation. The dielectric elastomer was then laminated such that its holes overlapped with the talc-coated surface of the pretensioned dielectric elastomer. The excess part was cut off, and the bioluminescent liquid was injected through the silicone tube. To establish an electrical connection to the outside, the silicone tube was sealed with a tube connector (ARAM, mini-connector type I), with copper wires fixed with adhesive.

For characterization, the underwater bending DEA was operated with a DC voltage input from a DC–DC converter (XP-Power, CB101) controlled by a function generator (Matsusada Precision, eK-FGJ) and a stabilized power supply (KIKUSUI, PMX32-2QU). A camera (Nikon, Z5, lens: AF-S Micro NIKKOR 60 mm f/2.8G ED) was used to capture the displacement of the tip angle when the voltage was applied. This actuation angle measurement experiment was conducted in a light environment. The angle between the tip of the bent DEA and the vertical direction was used as the tip angle in the measurement, and the displacement from the initial angle was measured. During this experiment, 16 independent samples were measured, and the average was recorded.

### 4.9 Fabrication and Characterization of the Jellyfish Robot

The manufacturing process for the jellyfish robot was identical to that of the underwater bending DEA. The jellyfish robot comprised three dielectric acrylic elastomer (3M, VHB 4905) layers. The middle layer had holes consisting of 30 mm × 10 mm rectangles connected to each side of an equilateral triangle with side lengths of 10 mm. The outer shape was determined by collecting a 5 mm offset from the hole shape. The upper layer had the same shape as the middle layer, and a film (TOYOBO, Pyrene) was attached at the position corresponding to the hole in the middle layer, while a silicone tube (MonotaRO, MGJG-1 × 2) was fixed to the central hole using adhesive (Cemedine, SUPER XG). The lower layer was pretensioned by 105% in three directions, and talc was applied at the positions corresponding to the holes in the middle layer. These dielectric acrylic elastomers were laminated, and the bioluminescent fluid was injected through a silicone tube. The silicone tube connected to the robot was attached to a Y-shaped connector (ARAM, Mini-Connector Y-type). The other end of the Y-shaped connector comprised a tube for liquid injection and an electrical connection through a copper wire reinforced with a heat-shrinkable tube (MonotaRO, SCG2.0–1B). The end of the electrical connection tube was connected to a float made of a laser-cut styrene board, which was electrically connected to an enamel wire on the float.

In the operation experiments, the jellyfish robot was operated using a square-wave voltage input from a DC–DC converter (XP-Power, CB101) controlled by a function generator (Matsusada Precision, eK-FGJ) and a stabilized power supply (KIKUSUI, PMX32–2QU). The amplitude of the square wave voltage was 10 kV, the frequency was 0.5 Hz, and the duty ratio was 50%. A camera (Nikon, Z5, lens: AF-P DX NIKKOR 18–55 mm f/3.5–5.6G VR) was used to capture the swimming of the jellyfish robot when the voltage was applied. The exposure time was set to 2 s, with an f-stop of 3.5 and an ISO sensitivity of 25600. The experiments were conducted in a darkroom (As One, BBX-01X table-top darkroom) shielded by a blackout curtain (ETSUMI, blackout curtain for darkrooms).

### 4.10 Structural Data Visualization

The structural data of the crystal structure of the *Renilla reniformis* luciferase variant RLuc8-W121F/E144Q in complex with A coelenteramide (the postcatalytic enzyme–product complex) (PDBID:6YN2)^45^ and Venus (PDBID:1MYW)^46^ were obtained from the PDB. Molecular graphics were produced using UCSF ChimeraX, developed by the Resource for Biocomputing, Visualization, and Informatics at the University of California, San Francisco, with support from the National Institutes of Health R01-GM129325 and the Office of Cyber Infrastructure and Computational Biology, National Institute of Allergy and Infectious Diseases.^47^

## Supporting Information Available

Conductivity of liquid medium, effect of concentration of bioluminescent liquid and luciferin on luminescence, concentration of bioluminescent liquid in calibration curve, measurement of luminance excluding changes over time, and modeling of DES and DEA (PDF)
Jellyfish robot swimming underwater (MP4)
