# Supplementary material for: Electrically Driven, Bioluminescent Compliant Devices for Soft Robotics
Source: ACS Appl Mater Interfaces. 2025 Feb 11;17(7):11248–58. doi: 10.1021/acsami.4c18209 (PMC11843531; doi:10.1021/acsami.4c18209)
Supplement: Supplementary file 1 — am4c18209_si_001.pdf [file am4c18209_si_001.pdf]

Supporting Information

Electrically driven, bioluminescent compliant devices for soft robotics

*Kengo Kusama[1], Atsuro Oishi[*2], Hitoshi Ueno[2], Akihide Yoshimi[3], Miki Nagase[2], and Jun Shintake[1*]*

[1]Department of Mechanical and Intelligent Systems Engineering, The University of Electro-Communications, 1-5-1 Chofugaoka, Chofu, 182-8585 Tokyo, Japan

[2]Department of Anatomy, Kyorin University, 6-20-2 Shinkawa, Mitaka, 181-0004 Tokyo, Japan

[3]Cancer RNA Research Unit, National Cancer Center Research Institute, 5-1-1 Tsukiji, Chuo, 104-0045 Tokyo, Japan.

*E-mail: shintake@uec.ac.jp

*E-mail: aoishi@ks.kyorin-u.ac.jp

Conductivity of liquid medium.

The change over time of the resistivity of the liquid medium, which is the base of the bioluminescence liquid, was measured (Figure S1). The measurement method is the same as that of the resistivity of bioluminescence liquid described in Section 4.4.

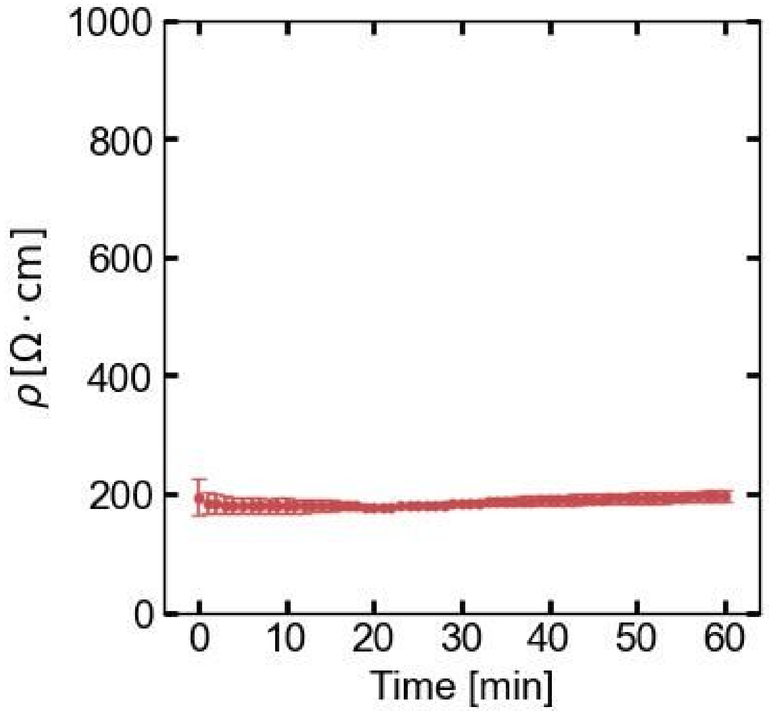

Figure S1. Resistivity of the luminescent solution over time.

Effect of concentration of bioluminescent liquid and luciferin on luminescence.

Original concentration of Bioluminescent liquid (100%) and 10 times (10% BL) and 100 times (1%BL) diluted bioluminescent liquid were mixed with each concentration of coelenterazine H and measured their luminescent kinetics in triplicates. The black, red, and blue arrows (Figure S2a) correspond to the colors of the corresponding kinetics graphs (Figure S2b).

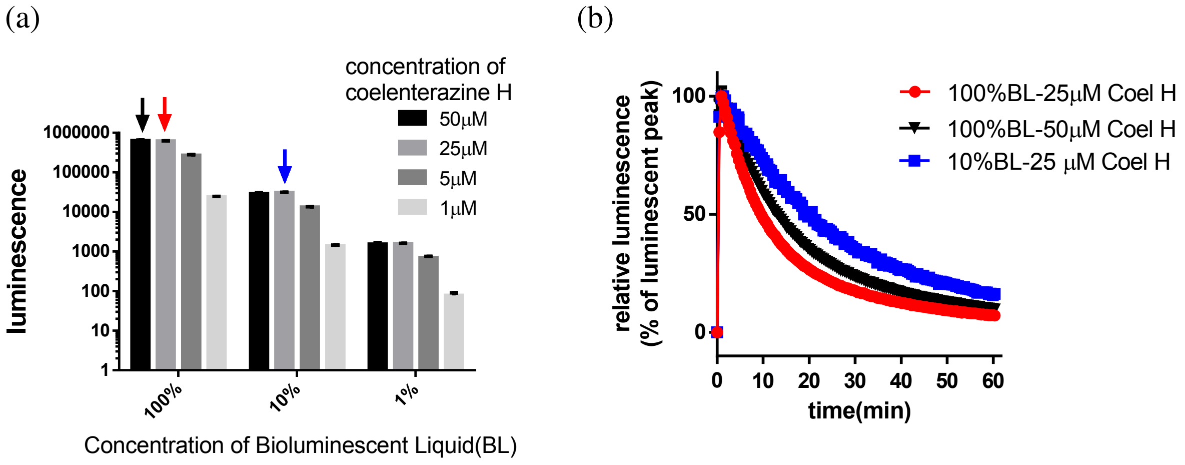

Figure S2. Effect of concentration of bioluminescent liquid and luciferin on luminescence. Relationship between luminescence intensity at its peak (a) and luminescence decay (b) for each bioluminescent liquid concentration and luciferin concentration.

Concentration of bioluminescent liquid in calibration curve.

In the calibration experiment, a luminescent solution was prepared by mixing luciferin and luciferase at a 1:40 ratio. By adding water to this luminescent solution, 10 different concentrations were prepared as indicated in Table S1. Micropipettes (ACCUMAX, Accumax Smart Pipettes) were used for the preparation.

Table S1. Mixture volume and concentration of the prepared bioluminescent solution.

	Bioluminescent liquid [μl]	Water [μl]	Total [μl]	Concentration of Bioluminescent liquid [%]
sample1	150.0	0.0	150.0	100.0
sample2	140.0	10.0	150.0	93.3
sample3	120.0	30.0	150.0	80.0
sample4	100.0	50.0	150.0	66.7
sample5	80.0	70.0	150.0	53.3
sample6	60.0	90.0	150.0	40.0
sample7	40.0	110.0	150.0	26.7
sample8	20.0	130.0	150.0	13.3
sample9	10.0	140.0	150.0	6.7
sample10	0.0	150.0	150.0	0.0

Measurement of luminance excluding changes over time.

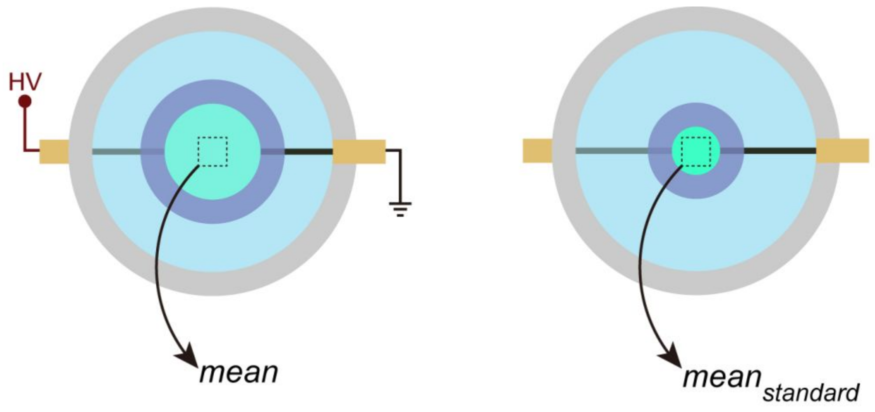

Figure S3. DEA and DES luminance income.

In the DES and DEA luminance change measurements in this paper, to mitigate the effects of temporal changes in luminescence, two experimental samples were prepared: one was subjected to operation, while the other was used as a luminance control. The luminance of the two samples was measured simultaneously (Figure S3): the luminance altered by the operation (*mean*) and the luminance obtained from the control ($mean_{standard}$). The luminance was normalized by dividing the luminance altered by the operation (*mean*) by the luminance from the control ($mean_{standard}$).

$$mean_{normalized} = \frac{mean}{mean_{standard}}$$

The initial normalized luminance ($mean_{normalized_0}$) was set to 100%, and subsequent normalized luminance ($mean_{normalized}$) were compared to this value.

$$Luminescence = \frac{mean_{normalized}}{mean_{normalized_0}}$$

Modeling of DES.

The capacitance values of the DES vary with uniaxial strain[1]. The initial capacitance of such a DES at zero applied strain is expressed as

$$C_0 = \varepsilon_0 \varepsilon_r \frac{w_0 l_0}{d_0},(1)$$

where w_{s0} denotes the initial width (perpendicular to the direction of strain) of the electrodes, and l_0 denotes the initial length (parallel to the direction of strain) of the electrodes. The deformation of the DES under unidirectional strain (□) is expressed as

$$w = \frac{w_0}{\sqrt{\epsilon + 1}}, l = l_0(\epsilon + 1), d = \frac{d_0}{\sqrt{\epsilon + 1}},(2)$$

where w and l denote the width and length of the electrodes, respectively. Using (1) and (2), the capacitance of the sensor as a function of uniaxial strain can be mathematically expressed as

$$C = \varepsilon_0 \varepsilon_r \frac{wl}{d} = \varepsilon_0 \varepsilon_r \frac{w_0 l_0}{d_0}(\epsilon + 1) = C_0(\epsilon + 1),(3)$$

(3) indicates that the response of the DES is proportional to the applied strain. Consequently, the gauge factor of the DES, defined in the following equation, is equal to one.

Additionally, assume that the luminance L is proportional to the thickness t. If the volume remains constant, the stretch ratios of length, width, and thickness are denoted as λ_1, λ_2 and λ_3, respectively, and satisfy the following relationship.

$$\lambda_1 \lambda_2 \lambda_3 = (4)$$

Since the stretch ratios in the width and thickness directions are equal, we set $\lambda_2 = \lambda_3 = \lambda$. Therefore,

$$\lambda = \frac{1}{\sqrt{\lambda_1}} (5)$$

Consequently, the thickness t can be expressed in terms of the initial thickness t_0 as follows.

$$t = \lambda t_0 = \frac{1}{\sqrt{\lambda_1}} t_0 (6)$$

Furthermore, because the luminance L is proportional to the thickness t, the luminance L exhibits the same behavior.

$$\frac{L}{L_0} = \frac{t}{t_0} = \frac{1}{\sqrt{\lambda_1}} (7)$$

Table S2. Parameters used for Modeling of DES.

Symbol	Value
l_0	20 mm
w_0	5 mm
d_0	0.5 mm
ε_t	8.85×10^{-12} F/m
ε_r	3.21

Modeling of DEA.

In DEAs, the dielectric membrane is pre-stretched. The thickness of this membrane at zero voltage is defined as

$$d = \left(\frac{1}{\lambda_p}\right)^2 d_0 (8)$$

where λ_p denotes the stretching ratio along the radial direction, and d_0 represents the initial thickness of the membrane. For simplicity, we assume that d and the membrane volume remain constant regardless of the applied voltage[2] and the presence of electrodes is disregarded in this analysis. Thus, using (8), the strain along the thickness direction can be expressed as

$$\epsilon_s = -\frac{\varepsilon_0 \varepsilon_r}{Y_1}\left(\frac{V}{d}\right)^2 (9)$$

where ε_0 denotes the permittivity of free space, ε_r represents the relative permittivity of the membrane, Y_1 denotes the Young's modulus of the membrane subjected to 200% strain, and V represents the applied voltage. Subsequently, the active area where the electrodes overlap under an applied voltage is obtained as

$$A = \frac{1}{1+\epsilon_s} A_0 . (10)$$

From (10), the areal strain of a circular DEA as a function of the applied voltage is then expressed as

$$S_A = \frac{A - A_0}{A_0} = \frac{\Delta A}{A_0} . (11)$$

Additionally, assume that the luminance L is proportional to the thickness t. The volume V of the electrode of the bioluminescent liquid is expressed as

$$V = At.(12)$$

Therefore,

$$t = \frac{V}{A}(13)$$

represents the thickness of the electrode. V is constant. Furthermore, since the luminance L exhibits the same behavior as the thickness t

$$\frac{L}{L_0} = \frac{t}{t_0} = \frac{\frac{V}{A}}{\frac{V}{A_0}} = \frac{A_0}{A}(14)$$

and from equation (10) it becomes

$$\frac{L}{L_0} = \frac{A_0}{\frac{1}{1+\epsilon_s}A_0} = 1 + \epsilon_s(15)$$

Table S3. Parameters used for Modeling of DEA.

Symbol	Value
A_0	78.54 mm^2
λ_p	2
Y_1	97.6 kPa
d_0	0.5 mm
ε_t	8.85×10^{-12} F/m
ε_r	3.21

Modeling of waterproof bending DEA.

For the waterproof bending DEAs, the actuated deformation (bending angle θ) is determined by calculating the local minimum of the actuators' potential energies, including strain, electrostatic, and bending energy[3–6]. The bending DEA in this study consists mainly of three layers of elastomeric membranes. The dielectric membrane is pre-stretched in the length direction, forming a bending shape as a result of the equilibrium of the total potential energy. Assuming the neutral plane of bending is in the middle of the substrate's thickness, the effective length of the dielectric membrane that contributes to the actuation is expressed as:

$$l = l_p - \frac{1}{2}(d_0 + d_t + d_m)\theta, (16)$$

where l_p represents the length after pre-stretch, d_t represents the thickness of the top layer, and d_m denotes the thickness of the middle layer. The stretch ratios of the membrane in the length (λ_1), width (λ_2), and thickness (λ_3) are then expressed as:

$$\lambda_1 = l/l_0, \lambda_2 = 1, \lambda_3 = 1/\lambda_1, (17)$$

where l_0 denotes the initial length of the membrane (l_t/λ_p and λ_p represent the pre-stretch ratio). (17) assumes the incompressibility of the membrane ($\lambda_1\lambda_2\lambda_3 = 1$), and pure-shear deformation ($\lambda_2 = 1$), as the bending DEA in this study experiences relatively low strain (< 10%). Using the Neo-Hookean material model[7], the strain energy of the dielectric membrane is expressed as:

$$U_{str} = l_0 w_0 d_0 \frac{Y_2}{6}\left(\lambda_1^2 + \lambda_2^2 + \lambda_3^2 - 3\right), (18)$$

where w_0 represents the initial width of the membrane and Y_2 denotes the Young's modulus of the membrane subjected to 10% strain. Substituting (16) and (17) into (18) allows the calculation of the strain energy.

The electrostatic energy of the actuator U_{ele} is defined as:

$$U_{ele} = -\frac{1}{2}CV^2 = -\frac{\varepsilon_0 \varepsilon_r l_{ele} w_{ele} \lambda_1^2 V^2}{2d_0},(19)$$

where w_{ele} denotes the width of the electrode and l_{ele} denotes the length of the electrode which is considered constant in (17). The sign of U_{ele} is negative as it considers the voltage-controlled case[3].

In the model, the bending energy is calculated for the top and middle layers. The bending energy of the top and middle layers is defined as:

$$U_{bend\_top} = \frac{1}{2}\frac{YI_t\theta^2}{l_p} = \frac{Yw_t d_t^3\theta^2}{24l_p},U_{bend\_middle} = \frac{1}{2}\frac{YI_m\theta^2}{l_p} = \frac{Yw_m d_m^3\theta^2}{24l_p},(20)$$

where Y represents the Young's modulus and $I_{t,m}$ denotes the second movement of inertia of the top and middle layers.

The total potential energy in the actuator is then given as the sum of (18)–(20)

$$U_{total} = U_{str} + U_{ele} + U_{bend\_top} + U_{bend\_middle},(21)$$

where θ is obtained by minimizing (21), which is equivalent to solving:

$$\frac{\partial U_{total}}{\partial \theta} = 0.(22)$$

Table S4. Parameters used for Modeling of waterproof bending DEA.

Symbol	Value
l_p	30 mm
λ_p	1.1
l_t	30 mm
w_t	20 mm
d_t	0.5 mm
l_m	30 mm
w_m	10 mm
d_m	0.5 mm
w_0	20 mm
Y_2	345 kPa
l_{ele}	30 mm
w_{ele}	10 mm
d_0	0.5 mm
Y	450 kPa
ε_t	8.85×10^{-12} F/m
ε_r	3.21

References

(1) Shintake, J.; Piskarev, Y.; Jeong, S. H.; Floreano, D. Ultrastretchable Strain Sensors Using Carbon Black-Filled Elastomer Composites and Comparison of Capacitive Versus Resistive Sensors. *Adv. Mater. Technol.* **2018**, *3* (3), 1700284. https://doi.org/10.1002/admt.201700284.

(2) Carpi, F.; De Rossi, D.; Kornbluh, R.; Pelrine, R.; Sommer-Larsen, P. *Dielectric Elastomers as Electromechanical Transducers*; 2008.

(3) Kofod, G.; Wirges, W.; Paajanen, M.; Bauer, S. Energy Minimization for Self-Organized Structure Formation and Actuation. *Appl. Phys. Lett.* **2007**, *90* (8), 081916. https://doi.org/10.1063/1.2695785.
(4) Shintake, J.; Rosset, S.; Schubert, B. E.; Floreano, D.; Shea, H. R. A Foldable Antagonistic Actuator. *IEEEASME Trans. Mechatron.* **2015**, *20* (5), 1997–2008. https://doi.org/10.1109/TMECH.2014.2359337.
(5) Rosset, S.; Araromi, O. A.; Shintake, J.; Shea, H. R. Model and Design of Dielectric Elastomer Minimum Energy Structures. *Smart Mater. Struct.* **2014**, *23* (8), 085021. https://doi.org/10.1088/0964-1726/23/8/085021.
(6) Shimizu, K.; Nagai, T.; Shintake, J. Dielectric Elastomer Fiber Actuators with Aqueous Electrode. *Polymers* **2021**, *13* (24), 4310. https://doi.org/10.3390/polym13244310.
(7) Kim, B.; Lee, S. B.; Lee, J.; Cho, S.; Park, H.; Yeom, S.; Park, S. H. A Comparison among Neo-Hookean Model, Mooney-Rivlin Model, and Ogden Model for Chloroprene Rubber. *Int. J. Precis. Eng. Manuf.* **2012**, *13* (5), 759–764. https://doi.org/10.1007/s12541-012-0099-y.
